# The role of immune cells in modulating chronic inflammation and osteonecrosis

**DOI:** 10.3389/fimmu.2022.1064245

**Published:** 2022-12-13

**Authors:** Jianrui Zheng, Zhi Yao, Lixiang Xue, Deli Wang, Zhen Tan

**Affiliations:** ^1^ Department of Bone and Joint Surgery, Peking University Shenzhen Hospital, Shenzhen, China; ^2^ Center of Basic Medical Research, Institute of Medical Innovation and Research, Peking University Third Hospital, Beijing, China

**Keywords:** osteonecrosis, inflammation, immune cells, osteoimmunology, cytokines, bone regeneration

## Abstract

Osteonecrosis occurs when, under continuous stimulation by adverse factors such as glucocorticoids or alcohol, the death of local bone and marrow cells leads to abnormal osteoimmune function. This creates a chronic inflammatory microenvironment, which interferes with bone regeneration and repair. In a variety of bone tissue diseases, innate immune cells and adaptive immune cells interact with bone cells, and their effects on bone metabolic homeostasis have attracted more and more attention, thus developing into a new discipline - osteoimmunology. Immune cells are the most important regulator of inflammation, and osteoimmune disorder may be an important cause of osteonecrosis. Elucidating the chronic inflammatory microenvironment regulated by abnormal osteoimmune may help develop potential treatments for osteonecrosis. This review summarizes the inflammatory regulation of bone immunity in osteonecrosis, explains the pathophysiological mechanism of osteonecrosis from the perspective of osteoimmunology, and provides new ideas for the treatment of osteonecrosis.

## 1 Introduction

Osteonecrosis is the death of bone and marrow cells as a result of chronic inflammation. Continuous stimulation by various adverse factors induces an immune response that, if unchecked, creates a chronically inflamed microenvironment that inhibits bone regeneration and repair. Osteonecrosis can be triggered by drugs, alcoholism, presence of sickle cell disease, or treatment with radiotherapy or chemotherapy (1–4). Osteonecrosis can occur in many parts of the body, especially around the joints, causing the collapse of mechanically encumbered subchondral bone and secondary osteoarthritis, which in turn causes pain and dysfunction that seriously affect the patient’s quality of life and eventually require surgery (1, 5–9). Each year, 20,000-30,000 new cases of osteonecrosis of the femoral head (ONFH) are diagnosed in the United States (10, 11) and about 150,000 cases of osteonecrosis in China (10, 12). Among cancer patients who received zoledronic acid for three years, the incidence of bisphosphonate-related osteonecrosis of the jaw is approximately 1.3% to 3.2% (13). As osteonecrosis can be a slow, progressive disease, its cumulative, long-lasting consequences place a significant burden on society, especially as populations around the world live longer.

The original intention of inflammation is to remove harmful stimuli or pathogens and promote tissue repair. The inflammatory response helps recruit factors that remove necrotic bone and intramedullary tissue. Indeed, bone injury causes an inflammatory response in bone tissue that is necessary for repair. Pro-inflammatory chemokines are secreted from injured tissues to recruit macrophages, neutrophils and other immune cells to remove harmful stimuli and regulate the resolution of inflammation. Bone marrow mesenchymal stem cells are also recruited to initiate bone repair (14, 15). Under normal conditions, the inflammatory response needs to dissipate in order to give way to regenerative processes. Otherwise, inflammation can become prolonged and thus impair tissue regeneration. In osteonecrosis, the persistence of harmful factors stimulates local immune cells to continuously secrete inflammatory factors, prolonging inflammation until it becomes chronic and impairing bone repair (16–18).

Osteoimmunology is an academic discipline that studies the interactions between bone cells (e.g., osteoblasts, osteoclasts, bone marrow mesenchymal stem cells) and immune cells (e.g., macrophages, T cells, B cells, neutrophils, dendritic cells) in the same microenvironment (19–22). These interactions are mediated by cytokines and signal transduction pathways. In the past, osteonecrosis was considered to result from the death of osteoblasts and osteocytes as well as abnormal activation of osteoclasts. However, studies have found a close link between abnormal immune responses and immune cell infiltration in osteonecrotic tissues, which show signs of uncontrolled inflammation (23–28). How various immune cells regulate inflammation in osteonecrosis has not been fully elucidated. This review summarizes current knowledge about the regulation of inflammation in osteonecrosis, and how immune cells perpetuate or abrogate osteonecrosis. In this way, the review elaborates the pathophysiological mechanism of osteonecrosis from an immunological perspective.

## 2 Uncontrolled inflammation leads to the failure of bone repair in osteonecrosis

**Figure 1:**
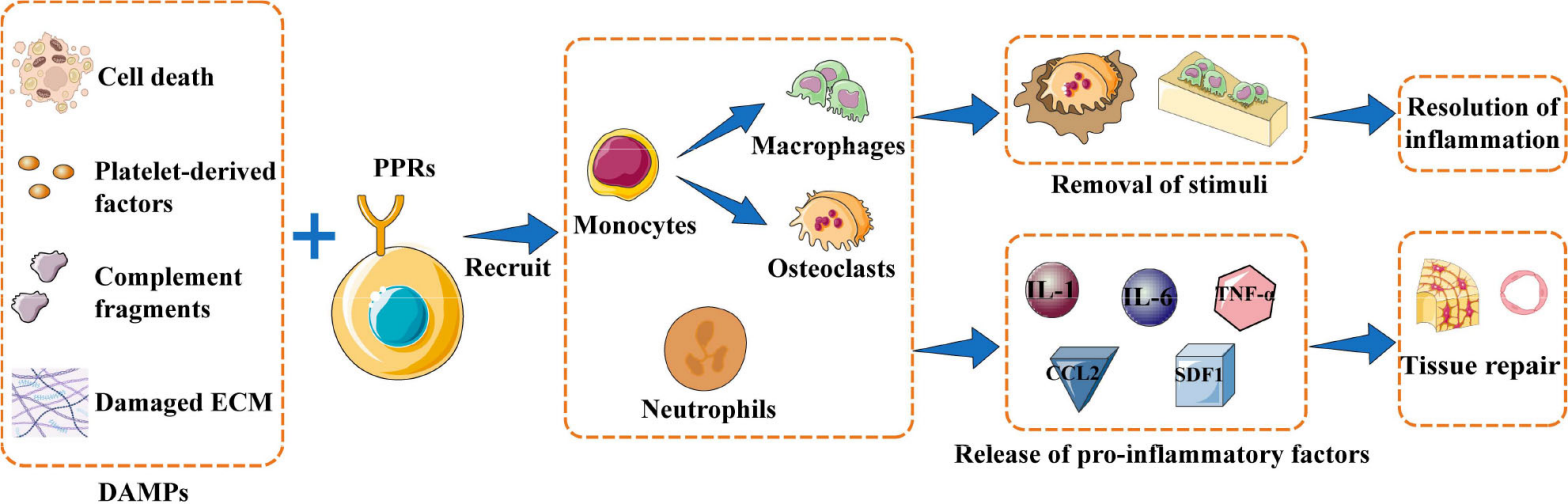
Inflammation initiates bone repair. When bone injury occurs, damage-associated molecular patterns (DAMPs) are recognized by pattern recognition receptors (PRRs) expressed on the surface of local cells. These cells are activated to release inflammatory factors that recruit immune cells, which can phagocytose bone fragments and cell debris or produce pro-inflammatory factors to recruit mesenchymal stem cells and initiate osteogenesis and angiogenesis. The overall result is resolution of inflammation and new bone tissue. Abbreviations: CCL2, C-C motif chemokine ligand 2; ECM, extracellular matrix; IL-1, interleukin 1; IL-6, interleukin 6; PMN, polymorphonuclear leukocytes; SDF1, stromal cell-derived factor 1; TNF-α, tumor necrosis factor–alpha.

The healing process after bone injury can be divided into three general stages: inflammation, callus formation, and remodeling (18). Bone injury results in death of bone cells and bone marrow cells, release of platelet-derived factors and complement fragments, and damage to the extracellular matrix. The net effect is that endogenous molecules act as damage-associated molecular patterns (DAMPs) that are recognized by pattern recognition receptors (PRRs) on local cells, which in turn activates inflammatory cascades (14, 18). Stimulated cells release cytokines and chemokines that induce immune cells to release even more pro-inflammatory factors, such as interleukin (IL)-1, IL-6, tumor necrosis factor (TNF)-α, C-C motif chemokine ligand 2 (CCL2) and stromal cell-derived factor 1 (SDF1) (14). This inflammatory response is a critical first step for eradicating harmful stimuli and removing cellular debris in order to help initiate the reconstruction of normal bone tissue. Inflammatory factors recruit neutrophils, macrophages, and osteoclasts to phagocytose and remove bone fragments and cell debris, while also activating mesenchymal stem cells to initiate osteogenic and angiogenic activities (14, 29–31). (**Figure 1**) The initial inflammatory response to bone injury usually dissipates within one week after the stimulus is removed. In the callus formation stage, bone marrow mesenchymal stem cells and osteoprogenitor cells participate in bone formation, which usually takes 1-3 months. The final remodeling stage takes months to years, during which new bone tissue is formed and shaped (14, 18).

**Figure 2:**
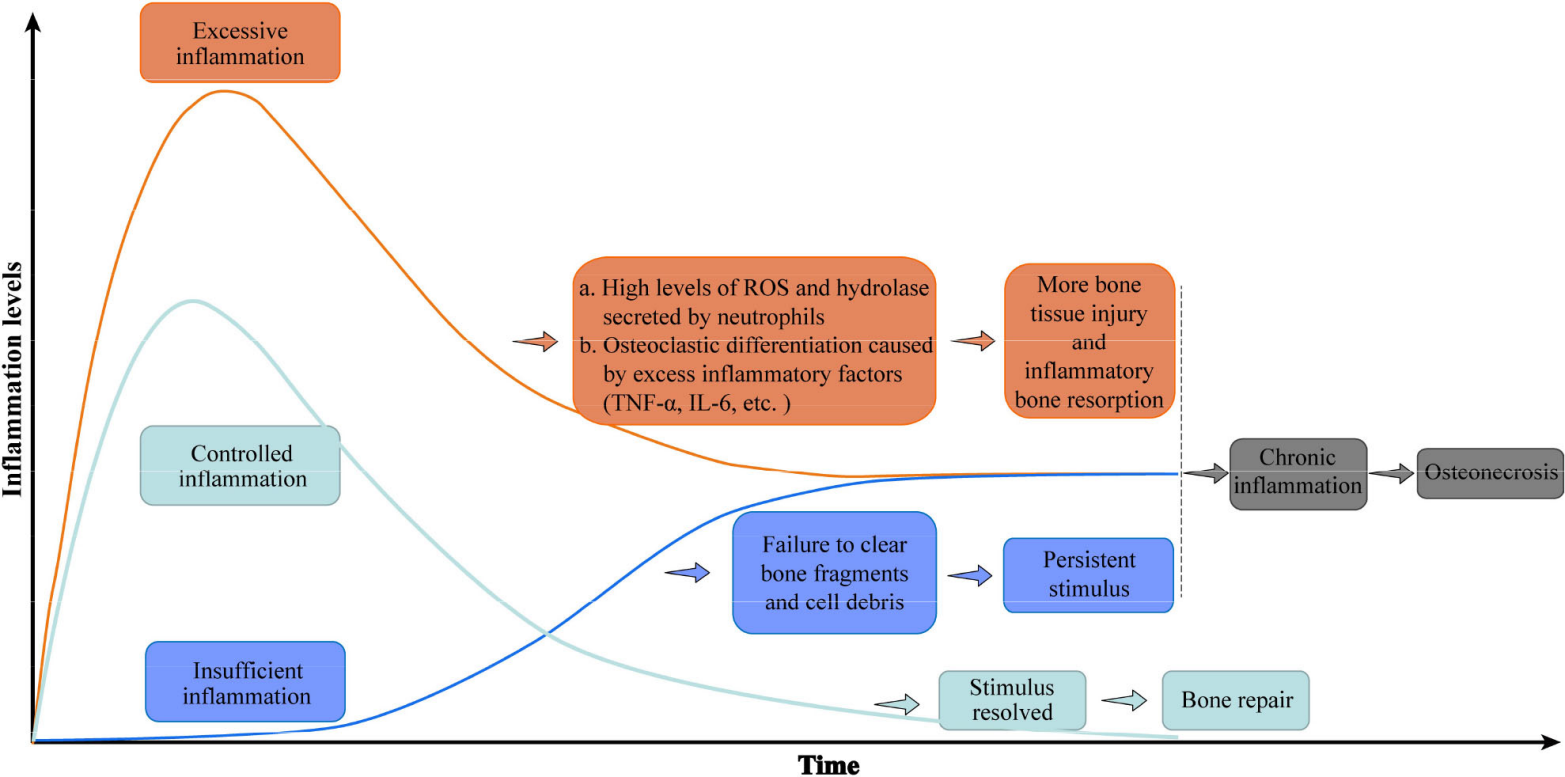
Uncontrolled inflammation promotes osteonecrosis. A controlled inflammatory response to bone injury activates immune cells to remove damaged tissue, then returns to baseline levels conducive to bone regeneration. Excessive inflammation maintains high levels of inflammatory factors that further destroy bone, while an insufficient inflammatory response fails to clear immune-activating factors. Either inflammatory disorder eventually leads to chronic inflammation and osteonecrosis. The green curve represents the change in the inflammatory level of controlled inflammation over time, while the orange and blue curves represent the inflammation level of excessive inflammation and insufficient inflammation, respectively. Abbreviations: IL-6, interleukin 6; ROS, reactive oxygen species; TNF-a, tumor necrosis factor–alpha.

Bone tissue repair depends on successful removal of harmful stimuli and suitable regulation of inflammation. An uncontrolled inflammatory response, either excessive or insufficient, is deleterious to bone repair. In the case of excessive inflammation, an overabundance of reactive oxygen species is produced, and proteases that damage the surrounding normal tissue are activated (32). Persistently high levels of inflammation inhibit the normal osteogenic response (16, 33). In the early stage of bone injury, transient signaling by TNF-α and IL-6 recruit the progenitors of osteoblasts required for bone regeneration, but persistently high levels of TNF-α and IL-6 inhibit osteogenesis and further damage bone tissue (14, 34). Excessive inflammation also stimulates osteoclast differentiation and activation, resulting in inflammatory osteolysis. Conversely, when the inflammatory response to bone injury is insufficient, local dead cell debris and bone debris are not completely removed, allowing DAMPs to persist in the microenvironment (14, 16). In either case, an excessive or insufficient inflammatory reaction eventually translates to chronic inflammation, which is the bridge between bone injury and osteonecrosis. Chronic inflammation hinders bone repair and regeneration following bone injury, which finally leads to osteonecrosis (10, 14, 16, 35–40). (**Figure 2**)

## 3 Immune cells and osteonecrosis

Chronic inflammation, the most prominent feature of osteonecrosis, occurs when inflammation prolongs resulting from the impaired resolution program (41–47). Persistent production of pro-inflammatory cytokines, progressive tissue injury and aberrant tissue remodeling are vital characteristics of this process (46, 48). In necrotic bone tissue, inflammatory cytokines/chemokines continuously recruit innate immune cells (macrophages, neutrophils, dendritic cells) and adaptive immune cells (T cells and B cells), which further release inflammatory factors in a positive feedback loop in order to amplify the overall inflammatory response (19, 20, 49). Furthermore, chronic inflammation excessively activates bone resorption and inhibits bone formation, driving osteonecrosis. In this way, disruption of the normal coordination between pro-inflammatory activation and anti-inflammatory silencing during bone repair may be the pathophysiological basis of osteonecrosis. Given that immune cells are the most important “modulators” of inflammation, elucidating how innate and adaptive immune cells regulate inflammation associated with osteonecrosis could provide insights into its pathogenesis and treatment.

### 3.1 Innate immune cells in osteonecrosis

#### 3.1.1 Macrophages

Macrophages are sentinels of the immune system. They identify and remove pathogens, kill target cells, present antigens, and regulate immune functions (50, 51). Macrophages differentiate mainly from monocytes and can be divided into classically activated macrophages (M1 phenotype) or alternatively activated macrophages (M2 phenotype) (50, 51). After bone injury, DAMPs released by bone and marrow cells recruit macrophages to the injured area and polarize them to the M1 phenotype, leading them to secrete pro-inflammatory factors such as TNF-α, IL-1β, and IL-6, which initiate and maintain inflammation (52–54). Four to seven days after bone tissue injury, secretion of anti-inflammatory factors such as tumor growth factor (TGF)-β and IL-10 into the microenvironment polarize M1 macrophages to the M2 phenotype. This shift in phenotype helps resolve inflammation, promotes secretion of mineralized matrix by bone marrow mesenchymal stem cells, and induces expression of the osteogenic factors alkaline phosphatase and osteocalcin, which enhance the osteogenic activity of osteoblasts. At the same time, anti-inflammatory factors inhibit osteoclast-mediated bone resorption, further supporting bone tissue repair (14, 55, 56). The regeneration and repair of bone tissue after injury depend on the precise order of macrophage polarization from M1 to M2.

In osteonecrosis, macrophages become locked in the M1 phenotype and continue to release inflammatory factors that exacerbate the initial tissue injury. Animal models of osteonecrosis showed high numbers of macrophage infiltration in areas with osteonecrosis, high ratio of M1 to M2 macrophages, and significant upregulation of pro-inflammatory factors TGF-β, IL-1β and IL-6 (57–59). Interestingly, a recent study of specimens from patients with non-traumatic ONFH also found that the main macrophage subset in the osteonecrosis area had the M1 phenotype, the local microenvironment was enriched with IL-1β and IL-6, and the ratio of M1 to M2 macrophages was significantly increased as osteonecrosis progressed (35). Inhibiting M1 macrophage polarization and reducing the M1/M2 ratio in femoral head and jaw reduced the secretion of local pro-inflammatory factors and the apoptosis of bone cells caused by inflammation, relieving steroid-induced osteonecrosis of the femoral head (SONFH) and bisphosphonate-related osteonecrosis of the jaw to some extent (60, 61). In addition, specifically regulating macrophage polarization from M1 to M2 to reduce the M1/M2 ratio downregulated the expression of pro-inflammatory factors in the osteonecrotic area, promoted the secretion of anti-inflammatory factors such as TGF-β and IL-10, reduced osteocyte apoptosis and promoted bone formation, allowing the regeneration and repair of necrotic bone tissue to a certain extent (62, 63). These findings suggest that M1 macrophage enrichment is an important osteoimmune feature of osteonecrosis and that targeting M1 macrophages is a promising therapeutic approach to treating osteonecrosis.

**Figure 3:**
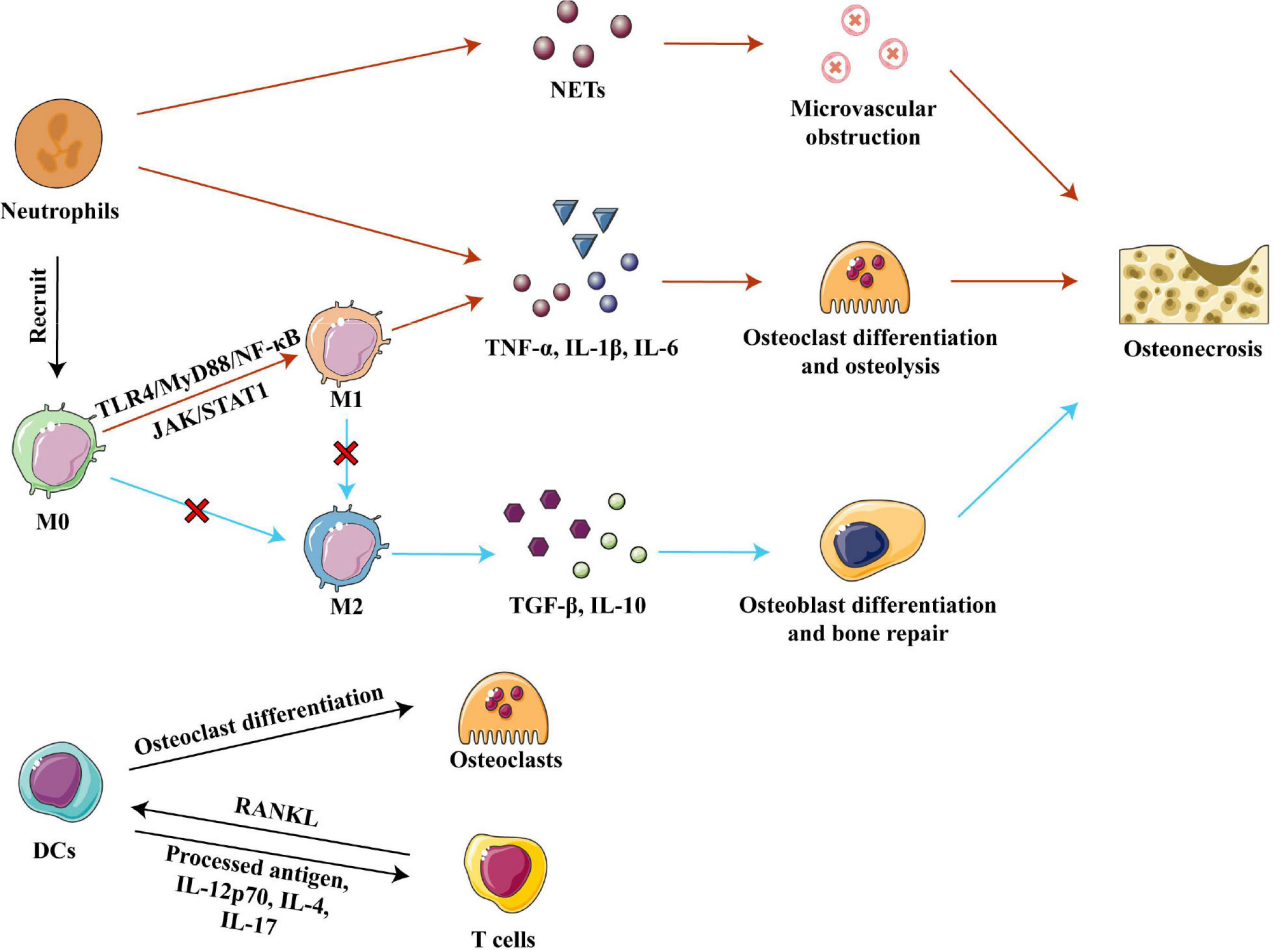
Innate immune cells in osteonecrosis. Neutrophils cause microvascular blockage and osteolysis by secreting, respectively, NETs and pro-inflammatory factors, resulting in osteonecrosis of the femoral head. Activation of the TLR4/MyD88/NF- κB and JAK/STAT1 pathways polarizes macrophages to the M1 phenotype, and they secrete inflammatory factors TNF-α, IL-1β and IL-6 to promote osteoclast differentiation and osteolysis. In osteonecrosis, macrophage polarization to the M2 phenotype is blocked, further impairing bone repair. DCs can differentiate into osteoclasts and participate in bone remodeling under the stimulation of RANKL secreted by T cells. DCs present processed antigens and secrete inflammatory factors that affect T cell differentiation. Abbreviations: DCs, dendritic cells; IL-1β, interleukin 1 beta; IL-4, interleukin 4; IL-6, interleukin 6; IL-10, interleukin 10; IL-12 p70, interleukin 12 p70; IL-17, interleukin 17; M0, Macrophages; M1, classically activated macrophages; M2, alternatively activated macrophages; NETs, neutrophil extracellular traps; RANKL, receptor activator of nuclear factor kappa-B ligand; TGF-β, tumor growth factor beta; TNF-α, tumor necrosis factor–alpha.

Strategies employed so far have targeted the upstream signaling pathways responsible for M1 polarization. Extracellular DAMPs released from injured bone bind to pattern recognition receptor toll-like receptor 4 (TLR4) on cell membranes and thereby activate the TLR4/MyD88/NF-κB signaling pathway, which promotes macrophage recruitment and M1 polarization (57, 64–66). Inhibition of TLR4/MyD88/NF-κB signaling *in vivo* by calycosin or TLR-4 inhibitor TAK-242 reduced the expression of various pro-inflammatory factors and promoted bone formation, effectively alleviating osteonecrosis in animals with SONFH and in bisphosphonate-related osteonecrosis of the jaw (33, 61, 66). On the other hand, some extracellular pro-inflammatory factors could activate the JAK/STAT1 pathway, which is another important pathway to promote M1 macrophage polarization (61, 67). Inhibition of the JAK/STAT1 pathway by using IL-17 inhibitor or curcumin inhibited the polarization of M1 macrophages in mice, significantly reduced the ratio of M1 to M2 macrophages, and prevented inflammatory-mediated apoptosis of osteocytes (60, 61). Therefore, methods to inhibit M1 polarization need to be further explored in order to develop potential therapeutic strategies for osteonecrosis. (**Figure 3**)

#### 3.1.2 Neutrophils

Neutrophils are derived from hematopoietic stem cells and mainly circulate in the peripheral blood. They have strong chemotactic and phagocytic properties (68). Once recruited to sites of bone injury, neutrophils secrete inflammatory and chemotactic mediators, such as IL-6 and MCP-1, which further recruit monocytes and macrophages (14).

The ability of neutrophils to promote inflammation in necrotic bone tissue is one of the important causes of osteonecrosis. Strong neutrophil infiltration occurs within one week of injury in ischemic osteonecrosis, but then neutrophil numbers taper off over time, although a low number persists in the microenvironment. These remaining neutrophils foster the occurrence and development of osteonecrosis through immune regulation of acute and chronic inflammation (49). The percentage of neutrophils in blood has been associated with the severity of SONFH, which may be because neutrophils promote osteoclast formation to accelerate bone resorption (23, 68). At the same time, neutrophils activated by necrotic tissue secrete neutrophil extracellular traps (NETs), which directly or indirectly induce the secretion of inflammatory factors (69–71). In ONFH patients, neutrophils are enriched in femoral head microvessels and the corresponding NETs interfere with blood flow, resulting in ischemic necrosis (69). Further studies in rats found that intravenous administration of neutrophils capable of forming NETs promoted the development of SONFH (69). Given the deleterious role of neutrophils in osteonecrosis, the removal of neutrophils may be a treatment for osteonecrosis. (**Figure 3**)

#### 3.1.3 Dendritic cells

In innate immunity, the main functions of dendritic cells (DCs) are phagocytosis and antigen presentation. DCs express a large number of PRRs, such as TLRs, C-type lectin receptors and NOD-like receptors, which recognize various DAMPs and pathogen-associated molecular patterns and quickly amplify local immune responses (72, 73). The contribution of DCs in osteoimmunology is two-fold: (1) DCs can differentiate into osteoclasts when stimulated by receptor activator of nuclear factor kappa-B ligand (RANKL) released from T cells, and the new osteoclasts participate in local bone remodeling; and (2) DCs can heavily influence the type of T cell responses by presenting processed antigen *via* major histocompatibility complex (MHC) class I and class II molecules, or by secreting pro- or anti-inflammatory cytokines such as IL-12 p70, IL-4, and IL-17 (45, 73, 74). (**Figure 3**) Which T-cell subtypes become involved and whether their net effect is to exacerbate or mitigate osteonecrosis will be discussed later in this review.

DCs serve as an important link between innate and adaptive immune responses by maintaining osteoimmune homeostasis. In contrast to the other innate immune cells, DCs may actually ameliorate osteonecrosis. In a mouse model, bisphosphonates impaired DC differentiation, maturation, migration and antigen presentation, ultimately inhibiting T cell activation and local immune responses, which translated to a higher risk of osteonecrosis of the jaw (75, 76). Two bioinformatic analyses showed decreased infiltration of activated DCs in ONFH (23, 27). These observations suggest that osteonecrosis may be due in part to DCs deficiency that impairs osteoimmune functions.

### 3.2 Adaptive immune cells in osteonecrosis

#### 3.2.1 T cells

T cells or T lymphocytes are an important component of cell-mediated adaptive immunity, and antigen-specific receptors on their surface can recognize antigens that antigen-presenting cells display on MHC complexes (77, 78). T cells can be divided into several subgroups based on their functions, and these subgroups can influence bone homeostasis. Various T cell subtypes work together to maintain the balance between osteogenic and osteoclastic metabolism by secreting osteoprotegerin (OPG) and RANKL or regulating the local inflammatory microenvironment, which in turn affects bone metabolism (77–79).

**Figure 4:**
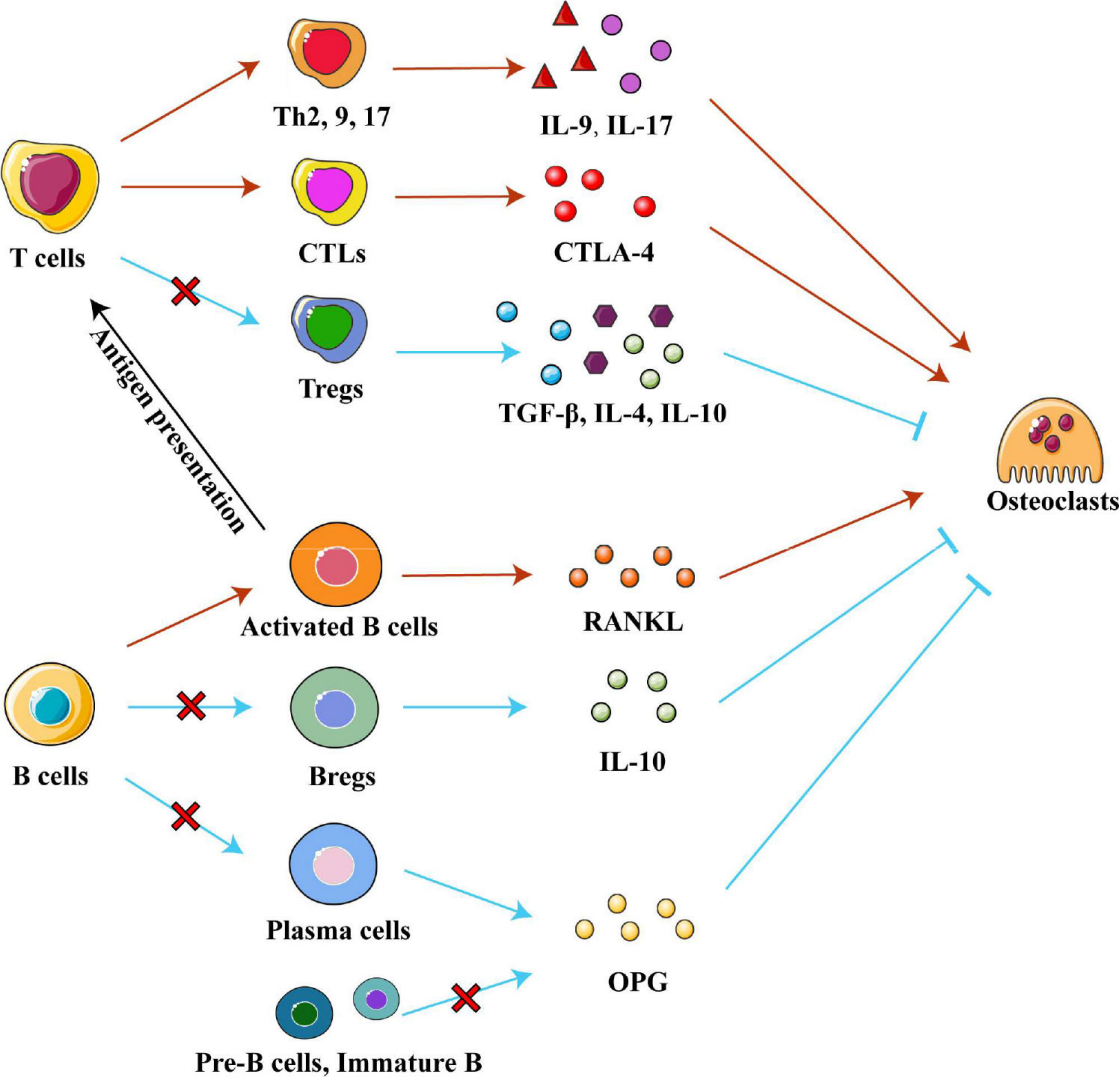
Adaptive immune cells in osteonecrosis. T cells can differentiate into the T helper cells (Th), cytotoxic T lymphocytes (CTLs) and regulatory T cell (Tregs) subtypes, which secrete various cytokines to influence chronic inflammation and osteoclast differentiation in different ways. Pre-B-cells and immature B cells are found only in bone marrow, while Bregs, plasma cells and activated B cells are recruited into osteonecrosis tissue. Activated B cells affect differentiation of T cell subtypes by presenting processed antigens and secrete RANKL to promote osteoclast differentiation. Bregs, plasma cells, Pre-B-cells and immature B cells secrete IL-10 and OPG respectively to inhibit osteoclast differentiation. Abbreviations: Bregs, regulatory B cells; CTLA-4, cytotoxic T lymphocyte-associated protein 4; IL-4, interleukin 4; IL-9, interleukin 9; IL-10, interleukin 10; OPG, osteoprotegerin; RANKL, receptor activator of nuclear factor kappa-B ligand; TGF-β, tumor growth factor beta.

Interestingly, T helper (Th) cells and cytotoxic T lymphocytes (CTLs) contribute to the progression of osteonecrosis, while regulatory T cells (Tregs) alleviate it. Th17 cells are enriched and activated in local tissues of ONFH and osteonecrosis of the jaw, and Th17 cells secrete IL-17 to maintain a chronic inflammatory microenvironment (80). IL-9 secreted by Th2, Th9 and Th17 cells upregulates inflammatory factors and enzymes related to cartilage degradation, promoting ONFH progression (42, 81). High numbers of CTLs infiltrate areas of osteonecrosis and contribute to it (24). They promote interactions between T cells and osteoclasts and enhance the activity of osteoclasts by secreting cytotoxic T lymphocyte-associated protein 4 (CTLA-4) (79). Conversely, Tregs may play a positive role in osteonecrosis, unlike Th and CTLs. The number of Tregs was found reduced in areas of osteonecrosis in mice (82). Further research found that Tregs secrete anti-inflammatory factors such as IL-4, IL-10 and TGF-β in non-traumatic ONFH in order to promote the resolution of inflammation while inhibiting osteoclast activity and osteolysis (79). Therefore, regulating the differentiation of T cells may be a strategy to treat osteonecrosis. (**Figure 4**)

#### 3.2.2 B cells

B cells or B lymphocytes secrete antibody molecules to initiate adaptive humoral immune responses and present antigens to activate specific T cell immunity (83, 84). B cells help maintain a normal bone microenvironment, and abnormal numbers of some B cell subtypes may be associated with osteonecrosis. Compared to healthy people, ONFH patients show significantly higher numbers of CD5+CD19+ B1 cells, CD86+CD19+ and CD95+CD19+ activated B cells, and CD27+CD95+CD19+ memory B cells in the blood (79, 85). Conversely, osteonecrotic tissue shows local decreases in the number of memory B cells and the total number of B cells (86). These observations emphasize the importance of B cells in maintaining the normal bone microenvironment and the ability of different B cell subtypes to influence the progression of osteonecrosis.

Different subtypes of B cells regulate bone metabolism by exerting different regulatory effects on osteogenic and osteoclast metabolism. Regulatory B cells (Breg) are a newly discovered subpopulation of B cells, which can secrete the anti-inflammatory factor IL-10 and inhibit osteoclast differentiation (83, 87, 88). An *in vivo* study found that low levels of Bregs led to low levels of IL-10 and activation of osteoclastic metabolism (88). In an *in vitro* study, regulating Breg differentiation reduced the levels of IL-6, IL-17 and TNF-α as well as promoted Treg differentiation (87, 88). In addition, OPG/RANKL system is another pathway through which B cells affect bone metabolism. Pre-B cells, immature B cells, and antibody-secreting B cells (plasma cells) inhibit osteoclast differentiation by producing copious amounts of OPG to block the RANK/RANKL system. (Indeed, this OPG production accounts for 40-60% of total OPG in the bone marrow.) On the contrary, activated B cells secrete RANKL under pro-inflammatory conditions to activate osteoclast formation (89–91). Boosting beneficial B cell subtypes over detrimental subtypes may be a treatment for osteonecrosis, which future studies should explore. (**Figure 4**)

## 4 Conclusion

During the development of osteonecrosis, necrotic bone damages local immune function, which leads to uncontrolled inflammation that creates a chronic inflammatory microenvironment, hindering bone regeneration and repair. This review summarizes the importance of immune cells and the regulation of their inflammatory responses in the pathogenesis of osteonecrosis on the basis of several original theories of osteonecrosis. It explains the pathophysiological mechanism of osteonecrosis from an immunological perspective according to the literature.

The immune system clearly exerts complex, pleiotropic effects on the development and severity of osteonecrosis. Abnormal infiltration of injured bone by M1 macrophages, neutrophils, and certain T cell subsets worsens disease by creating an abundance of pro-inflammatory factors, while DCs, Bregs and Tregs dampen immune responses by secreting anti-inflammatory and osteoclast-inhibiting factors. Despite these insights, we still do not understand the role of most immune cells in the progression of osteonecrosis. This will require making sense of how specific environmental cues influence the differentiation of immune cell subtypes and sub-lineages, and how these various subpopulations communicate with one another. The cellular heterogeneity in bone will make this work particularly challenging. Nevertheless, such research is quite important for the development of potential treatments for osteonecrosis.

## Author contributions

ZT conceived the manuscript. ZT and JZ drafted the manuscript. JZ designed the figures. ZY provided valuable comments. LX and DW revised the manuscript critically for important intellectual content. ZT performed manuscript review and final version approval. All authors contributed to the article and approved the submitted version.
